# Supplementary material for: In Vitro Evaluation of the Inhibitory Activity of Different Selenium Chemical Forms on the Growth of a Fusarium proliferatum Strain Isolated from Rice Seedlings
Source: Plants (Basel). 2021 Aug 20;10(8):1725. doi: 10.3390/plants10081725 (PMC8398910; doi:10.3390/plants10081725)
Supplement: Supplementary file 1 [file plants-10-01725-s001.zip › plants-1345860-supplementary/Table S1.pdf]

Table S1 . Liquid chromatography-inductively coupled plasma mass spectrometry (LC–ICP-MS/MS) conditions.

LC conditions	
**Instrument**	Agilent 1260 Infinity II
**Column 1 (anion exchange)**	Hamilton, PRP-X100, 250 x 4.6 mm, 5µm particlesize
**Mobile phase**	Ammoniumacetate 200mM, pH=5.2, 2% methanol
**Elution mode**	Gradient mode:<br>0-9 min 100% mobile phase A<br>9-20min 100%mobile phase B
**Flow rate**	1 mL min^{-1}
**Injection volume**	80 µL
**ICP-MS/MS conditions**	
**Instrument**	Agilent 8900
**Rf power (W)**	1550
**Argon gas flow rate (L/min):**	
**Plasma gas**	15.0
**Auxiliary gas**	0.90
**Nebulizer gas**	1.05
**Ion sampling depth (mm)**	7.0
**MS mode**	MS/MS
**Measured mass (m/z)**	78 (Q1) ⇒ 94 (Q2)
**Cell gas**	O_2, 25% (0.37 $mLmin^{-1}$)
