# Supplementary figures and images for: In Vitro Evaluation of the Inhibitory Activity of Different Selenium Chemical Forms on the Growth of a Fusarium proliferatum Strain Isolated from Rice Seedlings
Source: Plants (Basel). 2021 Aug 20;10(8):1725. doi: 10.3390/plants10081725 (PMC8398910; doi:10.3390/plants10081725)

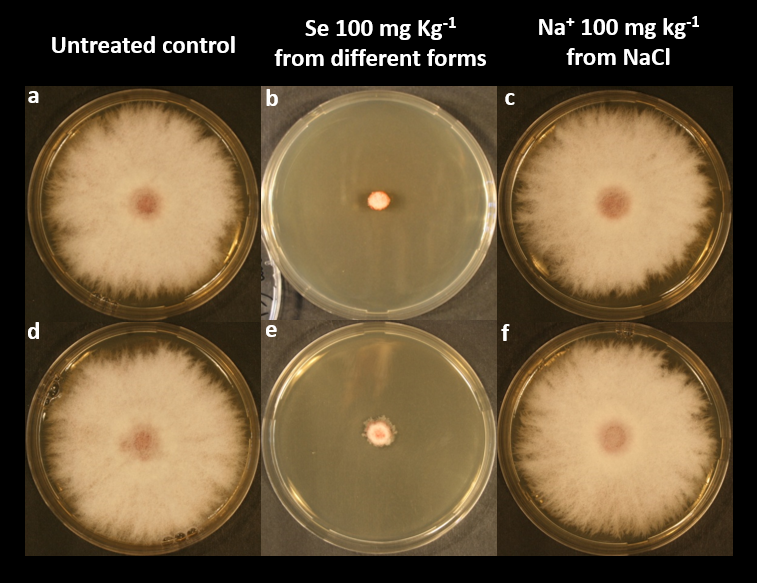

Supplement: Supplementary file 1 [file plants-10-01725-s001.zip › plants-1345860-supplementary/Figure S1.tif]

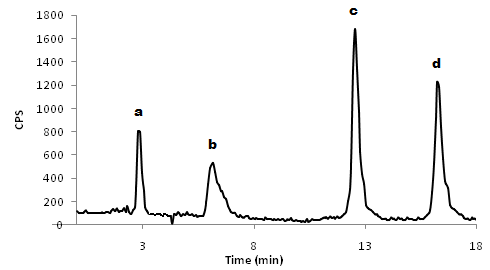

Supplement: Supplementary file 1 [file plants-10-01725-s001.zip › plants-1345860-supplementary/Figure S2.tif]
